# Supplementary material for: The Systems Biology Research Tool: evolvable open-source software
Source: BMC Syst Biol. 2008 Jun 29;2:55. doi: 10.1186/1752-0509-2-55 (PMC2446383; doi:10.1186/1752-0509-2-55)
Supplement: Additional file 1 — SBRT Archive. An archive of the current version of the Systems Biology Research Tool. [file 1752-0509-2-55-S1.zip › sbrt-1.4.0/doc/developers_guide/api/sbrt/shell/io/fba/package-summary.html]

sbrt.shell.io.fba


|  |  |  |
| --- | --- | --- |
| **Overview** \| **Package** \| Class \| **Use** \| **Tree** \| **Deprecated** \| **Index** \| **Help** | |  |
| **PREV PACKAGE**   **NEXT PACKAGE** | **FRAMES**    **NO FRAMES**     **All Classes** |  |


---

## Package sbrt.shell.io.fba

Provides classes and interfaces for reading and writing files relevant to
Flux Balance Analysis.

**See:**
  
          **Description**

| **Interface Summary** | |
| --- | --- |
| **FbaOptOutputFile** | This interface is used to represent FBA optimization output files. |

| **Class Summary** | |
| --- | --- |
| **CofoofSource** | This class is used to provide a source of column-oriented FBA optimization output files. |
| **ColumnOrFbaOptOutputFile** | This class is used to write column-oriented output files containing FBA optimization data. |
| **FluxCapInputFile** | This class is used to read files containing flux caps. |
| **FluxIntervalInputFile** | This class is used to read flux interval input files. |
| **RofoofSource** | This class is used to provide a source of row-oriented FBA optimization output files. |
| **RowOrFbaOptOutputFile** | This class is used to write row-oriented output files containing FBA optimization data. |
| **RxnNamExprSetInputFile<E extends MathExpr<S>,S>** | This class is used to read files containing a set of reaction name expressions. |
| **SerFbaOptOutputFile** | This class is used to write output files containing FBA optimization data as serialized maps. |
| **SfoofSource** | This class is used to provide a source of serialized FBA optimization output files. |

## Package sbrt.shell.io.fba Description

Provides classes and interfaces for reading and writing files relevant to
Flux Balance Analysis.

---


|  |  |  |
| --- | --- | --- |
| **Overview** \| **Package** \| Class \| **Use** \| **Tree** \| **Deprecated** \| **Index** \| **Help** | |  |
| **PREV PACKAGE**   **NEXT PACKAGE** | **FRAMES**    **NO FRAMES**     **All Classes** |  |


---
